# Drowning

**Published:** 1879-08

**Authors:** 


### DROWNING.

From *The Science Monthly* for June, 1879.

In cases of apparent drowning send immediately for medical aid, blankets and dry clothing, but proceed *instantly* to treat the patient on the spot in the open air, with the face downward, exposing the face, neck and chest to the wind, if the weather is not too cold, removing all tight clothing from the neck and chest, especially the braces. The points to be aimed at are, first and *immediately*, the restoration of breathing; and secondly, after breathing is restored, the promotion of warmth and circulation. The efforts to *restore breathing* must be commenced immediately and energetically, and persevered in for one or two hours, or until a medical man has pronounced life extinct. Efforts to promote *warmth and circulation*, beyond removing the wet clothes and drying the skin, must not be made until the appearance of natural breathing; for if circulation of the blood commence before breathing restoration to life is endangered.

